# A novel implant removal technique by endoscopy

**DOI:** 10.1186/s13018-018-0783-4

**Published:** 2018-04-06

**Authors:** Chang Heng Liu, Wen Lin Yeh, Ping Jui Tsai, Kuo Feng Fan, Hung Wei Cheng, Jian Ming Chen

**Affiliations:** 1 Department of Orthopaedic surgery, Chang Gung Memorial Hospital, Linkou Medical Center, #5, Fusing Street, Gueishan Township, Taoyuan County, 33305 Taiwan, Republic of China; 2 Bone and Joint Research Center, Chang Gung Memorial Hospital, Linkou Medical Center, Taoyuan, Taiwan; 3 Department of Athletic Training and Health, National Taiwan Sports University, Taoyuan, Taiwan; 4 College of Medicine, Chang Gung University, Taoyuan, Taiwan

**Keywords:** Arthroscopy, Endoscopy, Implant removal, Minimally invasive, Surgical technique

## Abstract

**Background:**

Routine implant removal after fracture healing remains controversial. However, it has been suggested that implant removal should be performed in cases of joint impingement, painful scar adhesion, and implant malposition. Entrance selection is relatively critical in patients with poor soft tissue conditions or sloughing coverage. We propose an innovative technique using endoscopy.

**Methods:**

Consecutive surgeries of endoscopic implant removal performed between 2005 and 2016 by a single experienced arthroscopic surgeon were included. Overall, 73 patients were enrolled; 44 were not eligible for inclusion and were excluded from the study.

**Results:**

Twenty-nine patients, including 32 surgical sites, were included. Twenty-four plates and 166 screws were removed using this technique. There were five complications during the follow-up period (range, 0.5 to 104 months; mean, 8.8), including one broken screw, one persistent knee joint contracture, and three wound dehiscence. There were no infections or neurovascular injuries.

**Conclusion:**

Implant removal using endoscopy is a minimally invasive surgery that ensures that the screw axis does not strip, and treats the intra-articular pathology concomitantly. This innovative technique may be considered as an alternative to the traditional open method in cases with good surgical indications.

## Background

The evolution of the treatment of patients with fractures is multifactorial. With the progression of biomaterials, biomechanics, fabrication, electronics, surgical facilities, and physician training, the use of fixators to treat patients with fractures has also dramatically progressed. Application of implants to the human body can be challenging in placement and removal. However, the concept of internal fixation dates back to the mid-1800s and remains the mainstay of fracture management. Internal fixators available in modern hospitals include the intramedullary nails, wires, pins, plates, and hybrid systems. The injury pattern, fracture location, and surgeon preference all dictate implant selection. Routine implant removal after fracture healing remains controversial [1–4]. Neurovascular injury, re-fracture, recurrent deformity, and wound infection are the possible complications of implant removal [5–7]. However, it has been suggested that implants should be removed in cases of painful scar adhesion, joint impingement with limited range-of-motion, prominent implants with skin tenting, and implant malposition. Because of patient differences, cultural factors also are important considerations in daily practice.

Surgeons generally use previous incisions when retrieving internal fixators, because a uniform entrance yields the same surgical field and better cosmetic outcome by avoiding a second scar. However, it is not always feasible to use the same entrance. Using the same entrance may be disastrous and result in serious complications in complicated soft-tissue problems and sloughing coverage [8–11]. Furthermore, minimally invasive procedures may be required in certain cases, and arthroscopy or endoscopy usually may be used. However, studies evaluating implant retrieval using endoscopy are limited [12–15]. The goal of this study is to propose a novel implant-retrieval technique using endoscopy under various conditions. We suggest that this innovative technique is useful and easy for most surgeons, with excellent patient satisfaction and significant clinical benefit.

## Material and methods

Consecutive cases that presented to our hospital (Chang Gung Memorial Hospital, Linkou) between 2005 and 2016 were enrolled in this study. Medical records were reviewed for surgical procedures, types of internal fixators, numbers of wounds needed for removal, wound healing condition, and complications. Patients who underwent knee ligament reconstruction or removal of prominent graft fixators were excluded. All surgeries were performed by a single experienced arthroscopic surgeon. A total of 73 cases were identified and 44 were excluded because they did not meet the criteria for inclusion. This retrospective review of medical records and radiographs was approved by the hospital’s institutional review board.

### Operative technique

Patients were positioned in either the supine or decubitus position for adequate surgical exposure while under general anesthesia. The plate contour and screw positions were palpated and marked on the skin with a marking pen. Portals were then designed according to different surgical sites and shapes of the internal fixators, which were facilitated for screw removal. One portal could generally be used to remove one to three screws; 0.5 cm for each portal was sufficient. First, a working space between the plate and scar tissue was made via blunt dissection followed by careful thermal shrinkage. The screw heads were visualized clearly under endoscopy, ensuring a match between the screwdriver and screw head. The screws were then removed sequentially under direct visualization. Gentle skin traction was needed to allow for the passage of the screw heads over the skin; skin extension was not necessary. Working and endoscopy portals were used alternately until all screws were removed. A number 18 needle was used to identify the screw head and the axis in difficult cases where the screws were in several different directions, including inter-fragmental or lag screws, and cases where screws could not be removed via the original portals, and the screw was removed via a new small stab wound. After removing all the screws, the plate was freely mobile under the subcutaneous space, and could be removed. Finally, the wounds were irrigated with normal saline, and closed with non-absorbable nylon sutures.

## Results

Twenty-nine patients (32 surgical sites) underwent endoscopic implant removal during the study period, including 15 men and 14 women with a mean age of 39 years (range, 20–72 years) at the time of the surgery. Surgical sites included 13 tibial plateaus, 7 lateral malleoli, 4 distal femurs, 3 clavicles, 2 tibial shafts, 2 distal tibias, and 1 medial malleolus. Twenty-six patients had fracture union, and mean time from index osteosynthesis to implant removal was 25.3 months (range 5–124 months). The other three patients were treated for chronic osteomyelitis-infected unions; they all had poor skin conditions due to the infection and repeated surgeries.

**Fig. 1:**
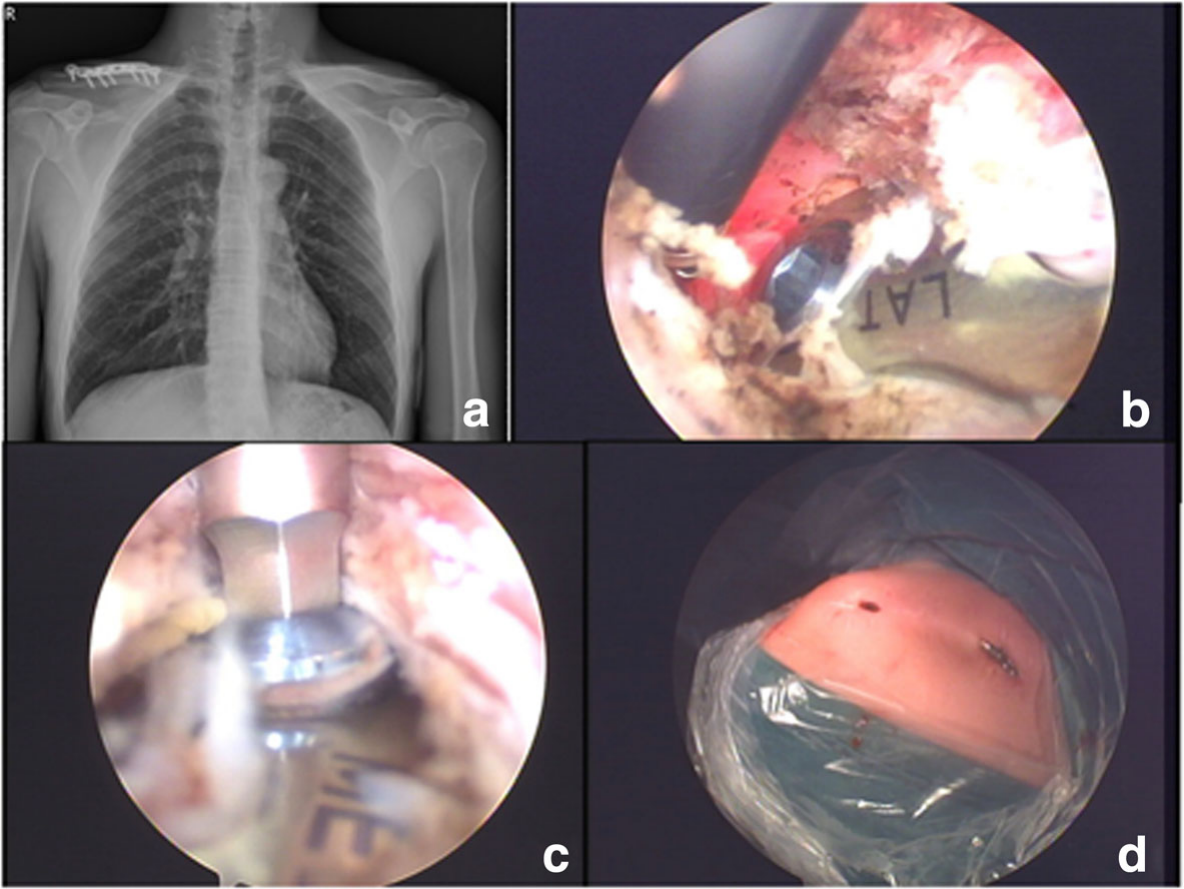
**a** A 34-year-old male received right clavicle open osteosynthesis with locking plate for 13 months, with bony union. **b**, **c** The plate and screws head could be visualized very clearly after debridement. **d** Totally seven screws and one plate were removed via this minimally invasive method

**Fig. 2:**
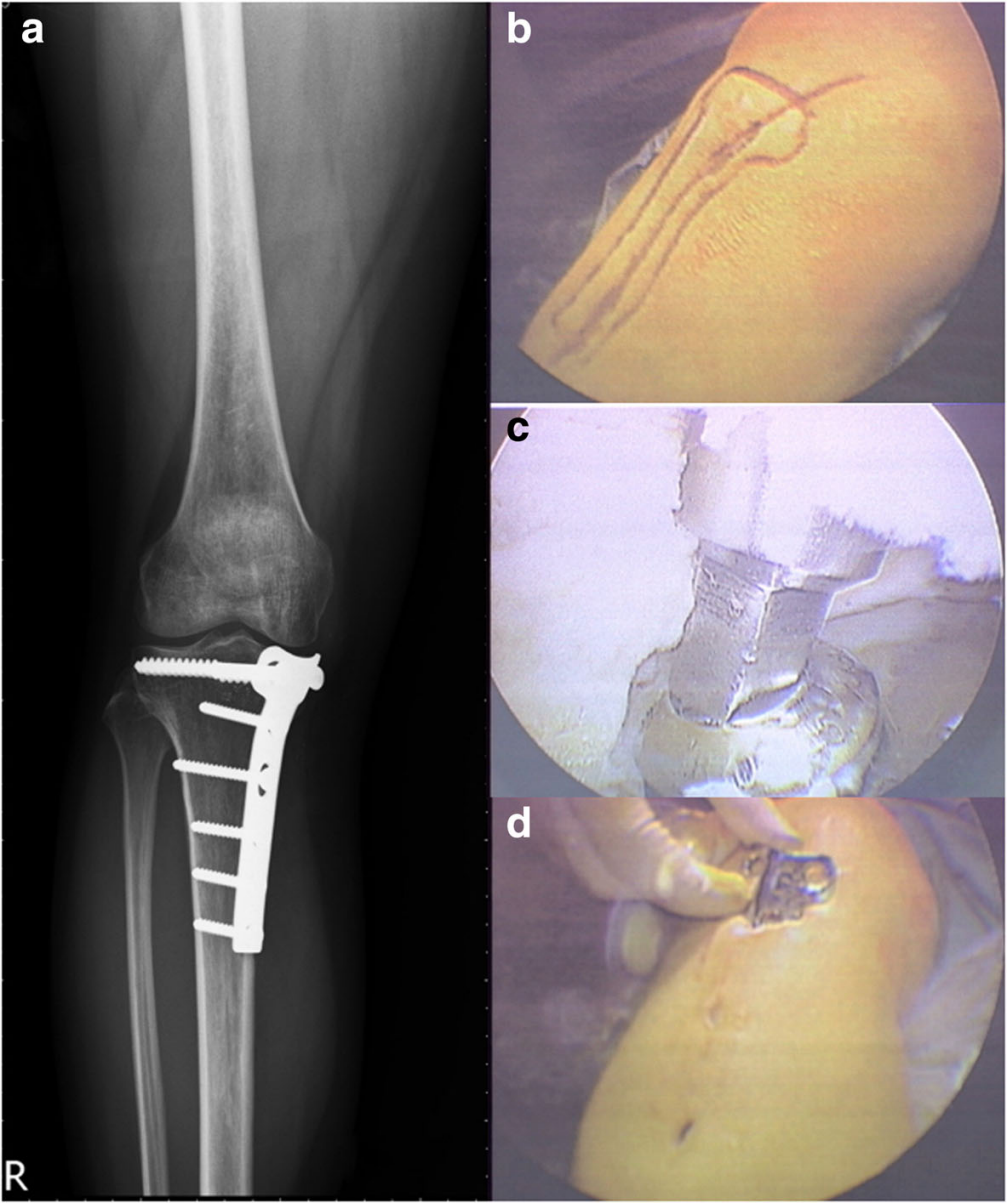
**a** A 42-year-old female suffered from right medial tibial plateau fracture, post T-type buttress plate fixation for 1 year. **b** The initial surgical wound and plate contour could be palpated directly, helping us to design the portal placement. **c** The screw axis could be seen very clearly to avoid striping screw head. **d** The plate was removed via the proximal wound

**Fig. 3:**
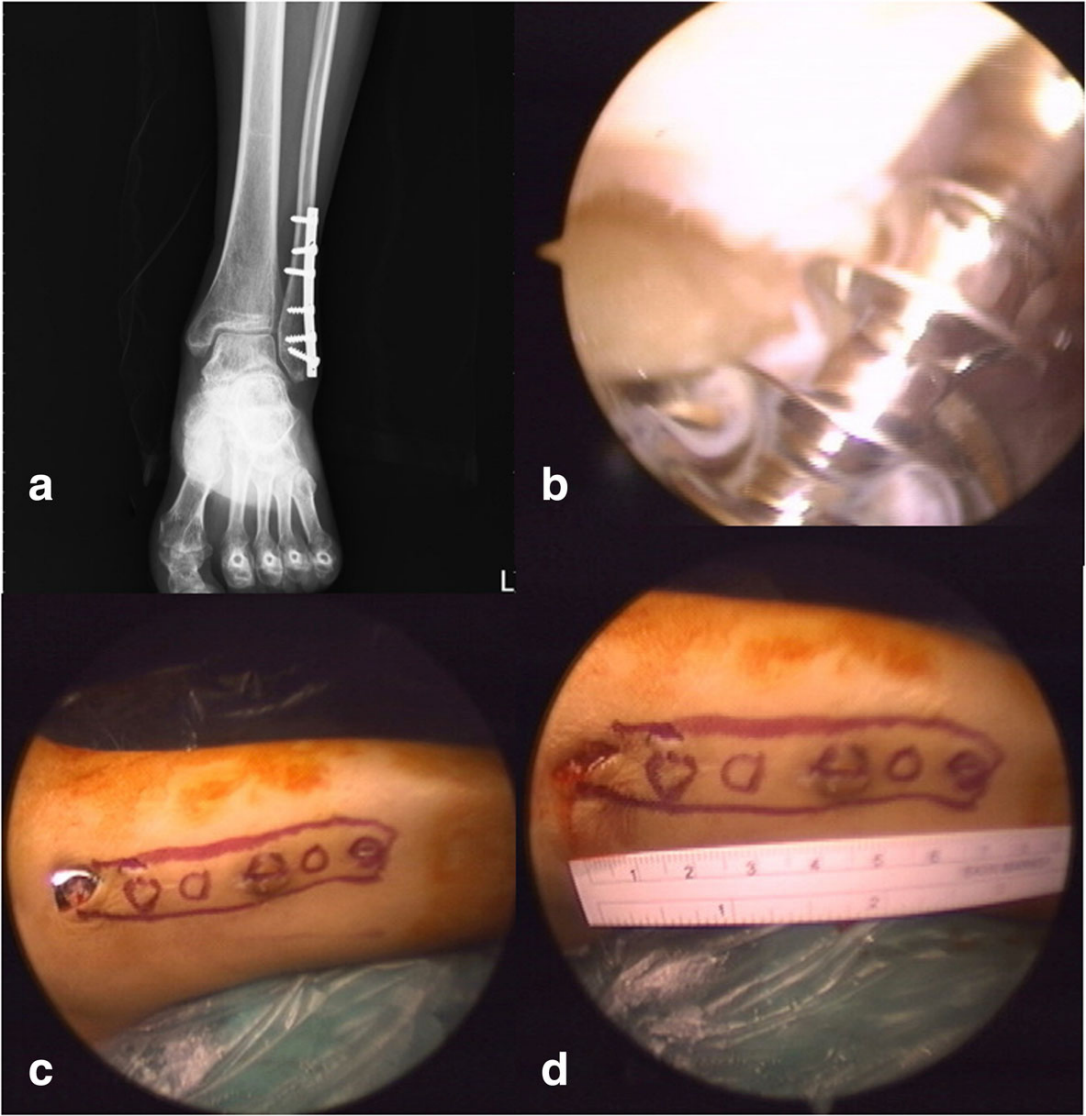
**a** A 34-year-old female was admitted from lateral malleolar semi-tubular plate removal and ankle arthroscopy examination. **b** The screws and plate were clearly identifiable after blunt dissection as well as scopic debridement of scar tissue. **c**, **d** Three small stab wounds rather than a traditional 7-cm large wound were enough for all the six screws and one plate removal

**Fig. 4:**
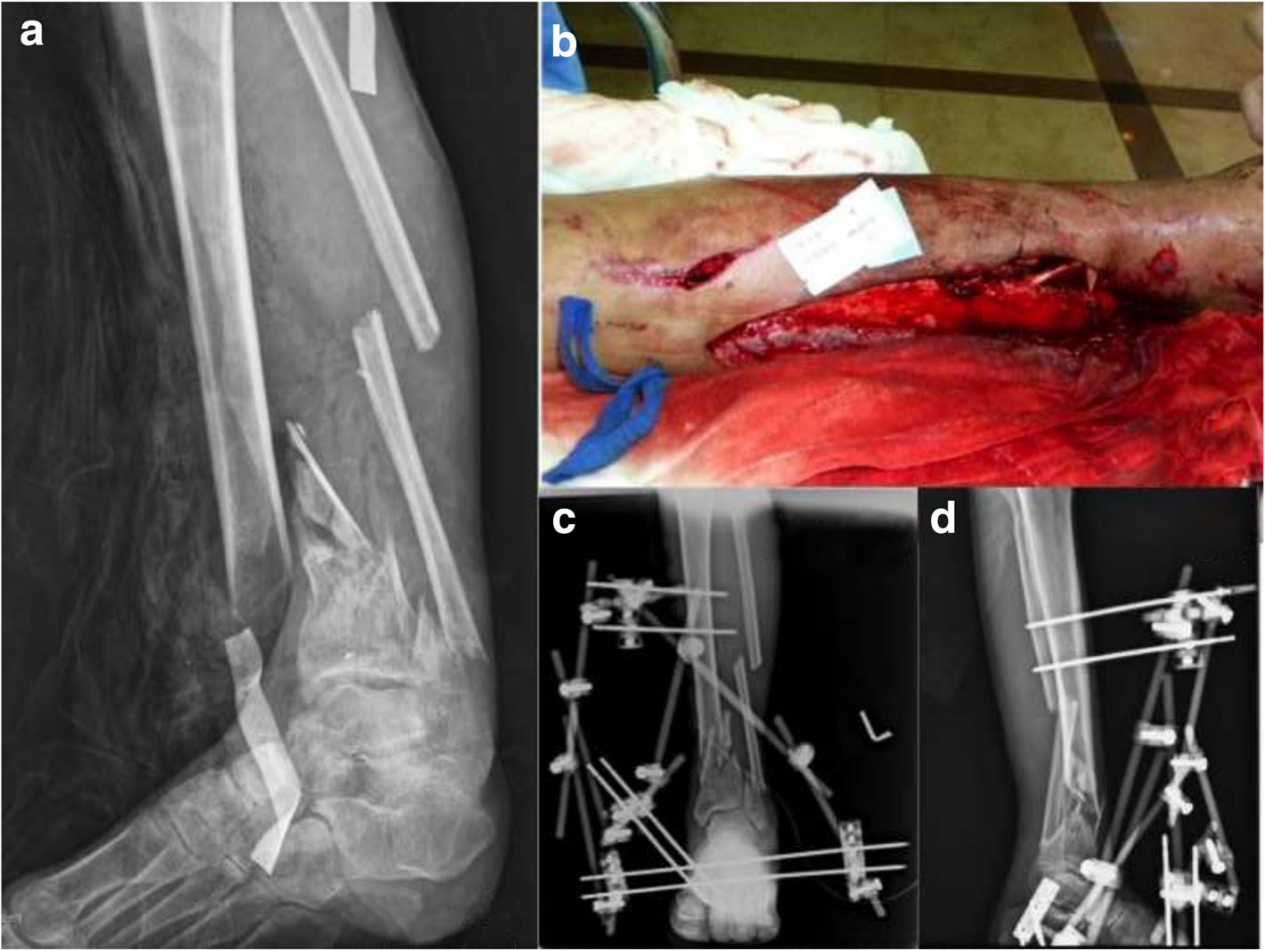
**a**, **b** A 40-year-old male suffered from polytrauma, including left distal tibiofibular fracture, Gustilo open type IIIb. **c**, **d** Staged operation was performed, and the patient received serial debridement, application of spanning external fixators, and then splint thickness skin graft reconstruction

**Fig. 5:**
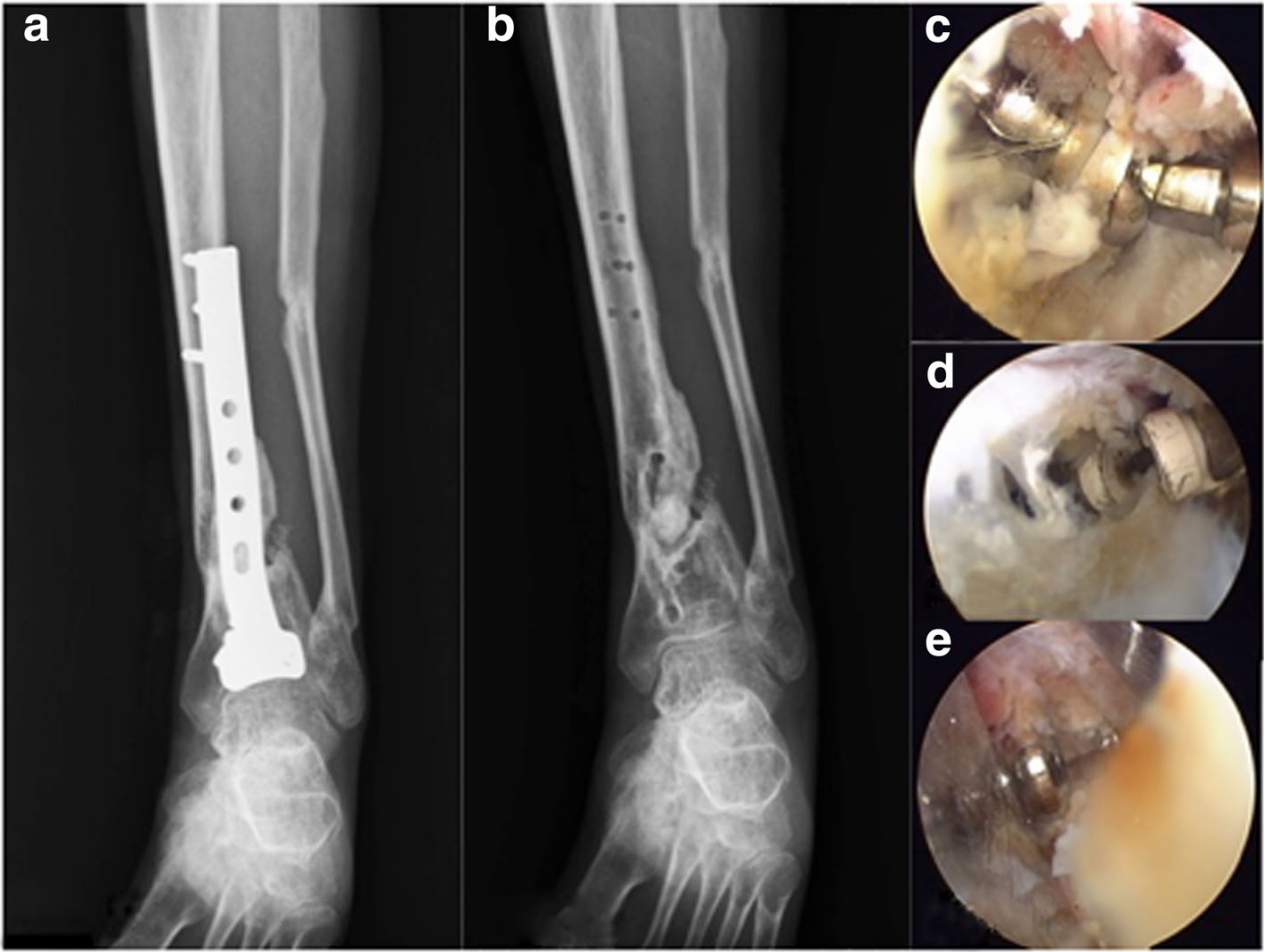
**a** Osteosynthesis with anterior tibial T-buttress plate was carried out 3.5 months later since injury. **b** There was fracture union 2.5 years post-operatively. **c** The implants were removed under assistance of endoscopy. **d**, **e** The soft tissue was hard without extensibility and the working space was relatively limited, so the electric dissector as well as mosquito or pean clamps were helpful to remove scar tissue and identify screws

A total of 24 plates and 166 screws were removed using this technique, including 6 traditional L- or T-shaped buttress plates, 6 semi-tubular plates, 5 locking plates, 4 dynamic compression plates, 1 reconstruction plate, 1 dynamic condylar screw, and one 95° L-plate (Figs. 1, 2, 3, 4, and 5). Mean surgical time, from skin incision to closure, was 49.8 min (range 20–133 min). Blood loss was minimal in all surgeries because most were performed using a tourniquet. The mean number of wounds for each case was 2.7 (range, 2 to 8). The average follow-up time was 8.8 months (range 0.5–104 months).

There were five complications, including one broken screw, three wound dehiscence, and one knee joint contracture. The broken screw was in the clavicle due to implant failure, and surgery was shifted to a mini-open method to remove the screw successfully. One wound dehiscence was in the lateral malleolus due to poor wound healing, which was resolved by repeated skin suture. The other two cases of wound dehiscence were chronic osteomyelitis of the tibial shaft, both of which received parenteral antibiotic therapy and wound care in the hospital for 7 and 12 days, respectively, and then were treated as outpatients. The last case was a fracture of the distal femur with knee contracture even after implant removal and arthroscopic release. This patient underwent another arthroscopic contracture release surgery 3 months later followed by regular physical therapy at a clinic.

## Discussion

Routine implant removal is usually not necessary after fracture healing [1–4, 16]. Symptomatic foreign body sensation with impaired limb function is the major indication for implant removal, followed by scar-related adhesion, local prominence, skin erosion, and mechanical. The traditional open approach is safe and familiar for most surgeons. However, additional removal surgery means further soft-tissue damage, which is associated with an increased risk for delayed wound healing and poor soft-tissue coverage of the bone, especially when entering from the previous incisions. With the advancement of arthroscopic techniques, the indication for minimally invasive surgery can be shifted from the intra-articular region to extra-articular sites (i.e., endoscopy). Endoscopic surgery provides all of the benefits of minimally invasive surgery and can be applied to implant removal with less soft-tissue damage and faster recovery [13]. Small stab wounds even meet the cosmetic concern. In our series, 29 patients (including 32 surgical sites) received the elective implant removal surgery, and only 1 case was complicated with surgery-related wound problem (the other 2 cases were related to chronic osteomyelitis). The wound complication rate was 3%, which was comparable to the surgical site infection rate of clean surgery (2%) [17] and much less than orthopedic implant removal surgery ever reported (12.2 to 20%) [9, 11, 18].

The application of endoscopy in implant removal is currently limited. Most cases involve the proximal humerus or shoulder joint region. Removing the proximal humerus plate and screws while concomitantly treating the glenohumeral joint or subacromial pathology via arthroscopy has been previously reported [12, 14, 19, 20]. Intra-articular adhesion arthrolysis, capsular release, and debridement of the subacromial impingement as well as early osteoarthritis and osteochondral lesion treatment can be performed simultaneously using this minimally invasive technique. It has also been shown to have promising results comparable to open procedures [13, 21]. However, implant retrieval from the proximal humerus is associated with an increased risk of axillary nerve injury [15]. Performing scar release with a shaver or electronic dissector before screw and plate removal especially places the axillary nerve at high risk for damage. Although the anatomical position of the motor branch of the axillary nerve is well understood [22], intraoperative fluoroscopy still may be required as a guide [15, 23]. A radiofrequency device with feedback function, rather than a shaver, for dissection also has been suggested.

Applying endoscopy in implant retrieval has several advantages, including minimal soft-tissue trauma and blood loss, as only a few small stab wounds are required [14, 23]. In addition, continuous saline irrigation during surgery lowers the risk of infection at the surgical site [15], while screw direction can be visualized under endoscopy, offering less chance of screw stripping, especially with locking screws. Finally, endoscopy can be shifted to the intra-articular region for arthroscopic examination, if indicated, after completing removal.

In our series, there were three cases of wound dehiscence, one in the lateral malleolus and two in the tibial shaft. The tibial shaft cases were both chronic osteomyelitis with initially poor skin condition. Therefore, there were still wound healing problems even with this minimally invasive technique. The other two complications—implant failure and persistent joint contracture—were not directly related to the surgical procedure. Regardless, the overall complication rate was quite low and acceptable, demonstrating that using endoscopy in implant removal is innovative and safe.

However, surgeons attempting this new technique must be very familiar with scope handling. Unlike routine joint arthroscopy, landmarks are usually deficient. Surgical planes should be created using blunt dissection by the surgeons themselves, and portal placement should be designed before skin incision based on the surgical site and implant type. In addition, the surgical orientation under endoscopy must be practiced several times with routine arthroscopy. Thus, we recommend mastering routine arthroscopy before attempting implant retrieval with endoscopy.

To our knowledge, this is the first article describing the application of endoscopy in implant removal. The indications for this minimally invasive technique include (1) retrieving implants from a relatively thin region, such as the clavicle, tibial plateau, tibial shaft, or lateral malleolus; (2) retrieving implants from a region with poor soft-tissue condition, such as an open fracture wound after skin grafting; (3) simultaneously retrieving implants and performing arthroscopic surgery, such as ankle joint examination after the removal of lateral malleolar implants; and (4) anticipated difficult wound closure by the traditional open method, such as hard scar tissue on the tibial plateau.

Using endoscopy to retrieve implants has never been published, and this is the strength of our study. Small patient numbers as well as lacking of control groups are the limitations. More cases with longer follow-ups are needed, and application of this technique in other surgical sites, such as the distal radius, femoral shaft, and pelvic region, could be studied in the future. With good patient outcomes, this new technique could be an alternative to the traditional open method.

## Conclusion

Implant retrieval using endoscopy is an innovative technique with all the advantages of minimally invasive surgery, including less soft-tissue damage, minimal blood loss, and rapid recovery. It is safe with a low complication rate, but surgeons should master arthroscopy before attempting this endoscopic technique. This technique may be an alternative to the traditional open method under good surgical indications.
